# Analysis of spatial differentiation and influencing factors of rural industrial integration efficiency in China

**DOI:** 10.1371/journal.pone.0336477

**Published:** 2025-12-11

**Authors:** Yuan Fang, Ying Yang

**Affiliations:** School of Finance and Economics, Qinghai University, Xining City, Qinghai Province, China; Universidad de Chile, CHILE

## Abstract

The integrated development of rural industries is a primary method to accelerate rural revitalization, a practical path to promote agricultural and rural modernization, and an important means to build a strong agricultural nation. Based on the super-efficiency EBM model, rural industrial integration efficiency in the east, central and west regions of 31 provinces (municipalities and autonomous regions) in China from 2012 to 2022 is measured, and the spatial differentiation, dynamic distribution and influencing factors of rural industrial integration efficiency are analyzed by using Dagum Gini coefficient, Kernel density estimation and Tobit model, respectively. The study shows that the core driving forces of rural industrial integration efficiency in the east, central and west regions are pure technical, comprehensive and pure technical efficiency, respectively; hyper-variable density is the main source of spatial differentiation; serious divergence in rural industrial integration efficiency in the east; there is regional heterogeneity in the impact of different factors on the efficiency of rural industrial integration. It is necessary to strengthen the structure of financial inputs, establish an efficiency assessment system, and enhance the infrastructure and public services.

## Introduction

In recent years, promoting the integrated development of rural industries has become an important issue of concern in the “agriculture, rural areas and farmers” policy, and China has introduced numerous policies to benefit and enrich the rural sector and strengthen it, creating a favorable environment for the integrated development of rural industries. Since 2015, when the No.1 Document of the Central Government first put forward the idea of “promoting the integrated development of primary, secondary and tertiary industries in rural areas”, the No.1 Document of the Central Government has made corresponding guidance and strategic planning for the integrated development of primary, secondary and tertiary industries in rural areas for 10 consecutive years until 2024. In addition, the “Standardization Action Plan for Rural Revitalization” in 2023 and the No. 1 Document of the Central Government in 2024 both propose to promote the continuous development of rural industrial integration. Since ancient times, China has been a large farming country, “agriculture is the foundation of the state, the foundation of the state peace”, only the “agriculture, rural areas and farmers” stable, in order to the world peace, but at present, the “agriculture, rural areas and farmers” in the development of the imbalance and inadequacy of the problem, which needs to be solved urgently, and the integration of rural industry is an important hand to solve the “agriculture, rural areas and farmers” problem. First, China’s rural industry involves “four chains”, i.e., industrial chain, supply chain, value chain and innovation chain, which are interconnected, and the study of a chain alone is not enough to fully reveal the current industrial development in rural areas of China, whereas through the integration of the rural industry, the industrial chain, supply chain, value chain and innovation chain can be put in the same perspective to study the whole chain of rural industries. which helps accelerate the upgrading of the whole rural chain and is crucial to the promotion of China’s rural industrial reform. Second, through the integration of rural industries, talent and capital can flow to the countryside, reduce the cost of agricultural production and expand the channels for farmers to increase their incomes, thereby resolving the imbalances and inadequacies in the development of the “agriculture, rural areas and farmers”, and realizing the goals of rural prosperity, agricultural efficiency and farmers’ increased incomes.

The integration of rural industries is a global research topic, with Western scholars primarily focusing on the integration of secondary and tertiary industries. The integration of rural industries in China is quite similar to that in Japan and South Korea. The concept of the “sixth industry” was proposed by Japanese scholar Naraomi Imamura in the mid-1990s. The level of integration of rural industries in Japan is relatively high, largely benefiting from the strength and organizational capability of the Japanese agricultural cooperatives. At the end of the twentieth century, against the backdrop of serious crises faced by rural and agricultural development in the country due to domestic and international environmental changes, South Korea drew on relevant experiences from Japan and launched the development and research of the ‘sixth industrialization’ based on its own practical circumstances. The level of integration of rural industries in South Korea is relatively high and is in a stage of continuous development.

The concept of industrial integration first originated from technological integration proposed by the American scholar [1]. Regarding the research on rural industrial integration, Western scholars have mainly studied the integration of secondary and tertiary industries, first proposing the concept of vertical integration in the field of agriculture [2]. Technological integration does not equate to industrial integration, and industrial integration should ultimately be achieved through market integration to realize comprehensive integration [3]. In contrast to European and American researchers who focus on vertical integration and technological convergence in agriculture, Asian scholars have focused on rural development. The concept of the “sixth industry” was put forward by Japanese scholar Naryashen Imamura in the mid-1990s [4]. Based on the six industries, Japanese scholars Sato Masayuki [5] and Shi Wu You Hong [6] conducted an analysis of the development status of Japan’s agricultural six-industry integration. The integration of the primary, secondary, and tertiary industries can achieve a seamless transition from production to sales through the extension of the industrial chain, allowing farmers to gain profits from the value added by the secondary and tertiary industries [7]. South Korea draws on the Japanese model and proposes the sixth industrialization and compound industrialization of agriculture, formulating policies to guide the integrated development of rural industries [8].

As far as domestic research is concerned, the connotation research, through the combination of the three industries within or between the three industries in a way to form a new mode of new business [9]. On the main body study, business subjects such as family farms, agribusinesses and professional cooperatives have become the backbone in the process of rural industrial integration [10]. Problems and countermeasures research, there are problems such as large differences between innovation subjects, poor technological and institutional innovation, and imperfect public services [11]. Insufficient collaboration in the industrial chain, bottlenecks in infrastructure construction, and low levels of digital literacy are constraining the integrated development of rural industries [12]. Although China has now made certain achievements in the process of rural industrial integration, the road of rural industrial integration and development has not been smooth or fast enough. Transforming the operation mode of new management subjects, the mode of rural industrial integration, and the mode of production organization are important driving forces for rural industrial integration [13]. On the study of integration level, the ecological niche width of rural industrial integration in the three southern border regions is centered on Kashgar City, showing a non-equilibrium situation, and the level of rural industrial integration is gradually increasing [14]. In the efficiency study, using the DEA method to measure the efficiency of integrated industrial development in rural areas of China, it has been concluded that the overall efficiency of rural industrial integration development in China is relatively low, with most provinces (municipalities and autonomous regions) experiencing varying degrees of efficiency loss [15]. The research on the efficiency of rural industrial integration using the BCC modified model under data envelopment analysis indicates that the overall efficiency level of rural industrial integration development in Gansu Province is relatively low, with significant differences in efficiency levels across various regions [16]. Select indicators such as the disposable income of rural residents, the capacity of rural power generation equipment, the total power of agricultural machinery, and the number of rural broadband access users, and utilize the Tobit model to conduct regression analysis on the factors influencing the development efficiency of the integration of the three industries in rural areas of China [17]. Using a fixed effects model, this study examines the impact of residents’ consumption demand, education expenditures, investment in rural infrastructure construction, and agricultural technological advancements on the efficiency of rural industrial integration [18].

A review of the literature reveals that, firstly, compared to the research on the mechanisms and levels of rural industrial integration in China, studies on the efficiency of rural industrial integration are relatively scarce. Secondly, in the papers studying the efficiency of rural industrial integration, a comparative analysis of the rural industrial integration efficiency across various provinces (municipalities, and autonomous regions) in China has been conducted, concluding that the efficiency of rural industrial integration in the eastern region of China is higher than that in the central and western regions. However, in reality, the eastern region inherently possesses significant advantages for rural industrial integration development due to factors such as policy support and economic development, which means that the starting points for the development of rural industrial integration efficiency in the eastern, central, and western regions are different. Thirdly, there is a limited use of the non-expected non-perspective super-efficiency EBM model to study the efficiency of rural industrial integration. Fourthly, in the research paper analyzing the factors affecting the efficiency of industrial integration in rural China, the influence factors of industrial integration efficiency in the rural areas of Eastern, Central, and Western China were not studied separately by region.

The contributions of this paper are as follows: Firstly, we measured the rural industrial integration efficiency across 31 provinces (municipalities, and autonomous regions) in China from 2012 to 2022. This included comparing the overall efficiency, pure technical efficiency, and scale efficiency of 11 provinces (municipalities, and autonomous regions) in the eastern region, 8 in the central region, and 12 in the western region. This approach effectively avoids the meaningless comparisons that could arise from the different starting points of rural industrial integration efficiency in the east, central, and west. Secondly, the application of the non-expected non-angles super-efficiency EBM model to study the efficiency of rural industrial integration effectively avoids the issue of calculating many provinces’ rural industrial integration efficiency as 1, which prevents further comparative analysis. Thirdly, conduct a study on the influencing factors of the efficiency of rural industry integration in the eastern, central, and western regions of China, comparing the different impacts of various influencing factors on different regions in a more targeted manner.

## Research methodology and data sources

### Research methodology

**Super-efficiency EBM model.** Traditional DEA models, such as the CCR model and the BCC model, are based on radial and angular references and have many prerequisites that can affect the accuracy of the measurement results to varying degrees. It is also due to the inability of the SBM model to measure both radial and non-radial input-output relationships, as well as the fact that the efficiency of rural industrial integration is measured as 1 in many provinces making it incomparable, while the super-efficiency model circumvents this problem [19]. Therefore, the improved non-expected non-angle super-efficiency EBM model was used to measure the efficiency of rural industrial integration. The specific model is as shown in formula (1.1):


$$\gamma^{*}=\min\frac{\theta -\varepsilon_{x}\frac{\text{1}}{\sum_{i=1}^{m}\varpi_{i}^{-}}\sum_{i=1}^{m}\frac{\varpi_{i}^{-}s_{i}^{-}}{x_{ik}}}{\phi +\varepsilon_{y}\frac{\text{1}}{\sum_{r=1}^{l}\varpi_{r}^{+}}\sum_{r=1}^{l}\frac{\varpi_{r}^{+}s_{r}^{+}}{y_{rk}}+\varepsilon_{b}\frac{\text{1}}{\sum_{t=1}^{q}\varpi_{t}^{b-}}\sum_{t=1}^{q}\frac{\varpi_{t}^{b-}s_{t}^{b-}}{b_{tk}}}$$ (1.1)

$$s.t.\left\{ \begin{array}{c} \sum_{j=1}^{n}x_{ij}\lambda_{j}+s_{i}^{-}=\theta x{}_{ik},i=1,...,m\\ \sum_{j=1,j\neq k}^{n}y_{rj}\lambda_{j}-s_{r}^{+}=\phi y{}_{rk},r=1,...,l\\ \sum_{t=1,j\neq k}^{n}b_{ij}\lambda_{j}+s_{t}^{b-}=\phi b{}_{tk},t=1,...,q\\ \sum_{j=1}^{n}\lambda_{j}=1,\lambda_{j}\geq 0,s_{i}^{-},s_{r}^{+},s_{t}^{b-}\geq 0,\theta \leq 1,\phi \geq 1 \end{array}\right.$$

Among them, γ^*^ indicates the efficiency of rural industrial integration, *x*_*ik*_, *y*_*rk*_ and *b*_*tk*_ represent the *k*-th input of the *i*-th decision-making unit, the *r*-th type of expected output and the *t*-th type of non-expected output, $s_{i}^{-}$, $s_{r}^{+}$ and $s_{t}^{b-}$ are the corresponding relaxation variables, $\varpi_{i}^{-}$, $\varpi_{r}^{+}$ and $\varpi_{t}^{b-}$represent the corresponding weights, *θ* and *φ* are the planning parameters of the radial part, ε∈[0,1] indicates the importance of the non-radial component when calculating the efficiency value of rural industrial integration.

**the Dagum Gini coefficient method.** Traditional coefficients of variation, Thei index and Gini coefficient, which are methods of examining regional differences, limited to the absence of cross-overlapping parts between the grouped samples, were unable to solve the problem of decomposition of regional differences, so the Dagum Gini coefficient method was proposed [20]. This method not only measures regional disparities, but also decomposes disparities from different sources so as to analyze the impact of different subgroups on the overall regional disparities [21]. Therefore, the Dagum Gini coefficient method was used to study the regional differences in the efficiency of rural industrial integration. The specific models are as illustrated in formulas (2.1) to (2.8):

Firstly, the Dagum Gini coefficient method is used to measure the overall gap in the efficiency of rural industrial integration. Subsequently, based on this result, it is decomposed into three components: intra-regional differences, inter-regional differences, and varying densities of super variance. The calculation formula is as follows:


$$G=\frac{\text{1}}{\text{2}n^{2}\overset{―}{y}}\sum {\mathrm{\backslash nolimits}}_{a=1}^{\theta }\sum {\mathrm{\backslash nolimits}}_{b=1}^{\theta }\sum {\mathrm{\backslash nolimits}}_{i=1}^{n_{a}}\sum {\mathrm{\backslash nolimits}}_{j=1}^{n_{b}}|y_{ai}-y_{bj}|$$ (2.1)

Among them, *θ* refers to the three groups (Eastern, Central, and Western) into which the 31 provinces (municipalities and autonomous regions) in China are classified, *y*_*ai*_ and *y*_*bj*_ respectively represent the rural industrial integration efficiency of any province (city, autonomous region) within region *a*(*b*) (a=1,...,θ;b=1,...,θ), *G* is the overall Gini coefficient for the efficiency of rural industrial integration. $\overset{―}{y}$ represents the average efficiency of rural industry integration across 31 provinces (municipalities and autonomous regions) in China. *n* represents the number of provinces (municipalities, autonomous regions), *n*_*a*_ and *n*_*b*_ represents the number of provinces (cities, autonomous regions) within the *a*(*b*) area respectively.

Decompose the Dagum Gini coefficient into three components: intra-regional differences, inter-regional differences, and super-density variation, satisfying the equation $G=G_{w}+G_{nb}+G_{t}$. Among them, *G*_*w*_ represents the contribution rate of spatial differences within the region, indicating the disparities in rural industrial integration efficiency among the 31 provinces (municipalities and autonomous regions) of China. *G*_*b*_ represents the contribution rate of spatial differences between regions, indicating the disparities in rural industrial integration efficiency among the three major regions of Eastern, Central, and Western China. *G*_*t*_ represents the contribution rate of ultra-variable density, indicating a residual Gini coefficient of the cross-effects of rural industrial integration efficiency among the three major regions.


$$G_{aa}=\frac{\text{1}}{2n^{2}\overset{―}{y}}\sum {\mathrm{\backslash nolimits}}_{i=1}^{n_{a}}\sum {\mathrm{\backslash nolimits}}_{j=1}^{n_{b}}|y_{ai}-y{}_{bj}|$$ (2.2)

$$G_{ab}=\frac{\text{1}}{n_{a}n_{b}({\overset{―}{y}}_{a}+{\overset{―}{y}}_{b})}\sum {\mathrm{\backslash nolimits}}_{i=1}^{n_{a}}\sum {\mathrm{\backslash nolimits}}_{j=1}^{n_{b}}|y_{ai}-y_{bj}|$$ (2.3)

$$G_{w}=\sum {\mathrm{\backslash nolimits}}_{a=1}^{k}G_{aa}p_{a}S_{a}$$ (2.4)

$$G_{nb}=\sum {\mathrm{\backslash nolimits}}_{a=2}^{k}\sum {\mathrm{\backslash nolimits}}_{b=1}^{a-1}G_{ab}(p_{b}s_{a}+p_{a}s_{b})D_{ab}$$ (2.5)

$$G_{t}=\sum {\mathrm{\backslash nolimits}}_{a=2}^{k}\sum {\mathrm{\backslash nolimits}}_{b=1}^{a-1}G_{ab}(p_{b}s_{a}+p_{a}s_{b})(1-D_{ab})$$ (2.6)

Among them, $p_{a}=\frac{n_{a}}{n}$, $s_{a}=\frac{n_{a}\overset{―}{y}}{n\overset{―}{y}}$, $D_{ab}=\frac{m_{ab}-s_{ab}}{m_{ab}+s_{ab}}$, *D*_*ab*_ indicates the relative impact of the integration efficiency of rural industries between regions and among them, *m*_*ab*_ indicates the differences in the efficiency of rural industry integration between regions, which can be understood as the mathematical expectation of the sum of all sample values where $y_{ai}-y_{bj}>0$ in both Region *a* and Region *b*; $s_{ab}$ represents the super-variable first-order moment, which can be understood as the mathematical expectation of the total of all samples in regions *a* and *b* where $y_{ai}-y_{bj}<0$. The formulas for calculating the two are:


$$m_{ab}=\int_{0}^{\infty }dF(y)\int_{0}^{y}(y-x)dF_{b}(x)$$ (2.7)

$$s_{ab}=\int_{0}^{\infty }dF_{b}(y)\int_{0}^{y}(y-x)dF_{a}(x)$$ (2.8)

Among them, *F*_*a*_ and *F*_*b*_ represent the cumulative density distribution functions of region *a* and region *b*, respectively.

**the kernel density estimation method.** Kernel density estimation is an unknown density function, which is a non-parametric estimation method that uses convolutional smoothing curves as weights to fit the probability density curves of the sample data [22]. Compared with the histogram of frequency distribution, kernel density is estimated to adopt continuous density functions to comprehensively reflect the dynamic evolution of variables. Therefore, the kernel density estimation method is used to study the distributional dynamics and evolution of the efficiency of rural industrial integration and development. The specific models are as illustrated in formulas (3.1) and (3.2):

This study employs Kernel Density Estimation to analyze the dynamic distribution and evolution of rural industry integration efficiency, with the following calculation formula:


$$f(x)=\frac{\text{1}}{Nh}\sum_{i=1}^{N}K(\frac{X_{i}-x}{h})$$ (3.1)

$$K(x)=\frac{\text{1}}{\sqrt{2\pi }}\exp(-\frac{\text{1}}{\text{2}}x^{2})$$ (3.2)

Among them, *K*(*x*)is the kernel function *f*(*x*) is a probability density estimate; *n* is the number of observations; *h* represents the bandwidth, a smooth parameter with a value greater than zero, indicating the smoothness and precision of the function curve; *X*_*i*_ represents the efficiency of rural industry integration in province (city, autonomous region) *i*; *x* represents the average efficiency of rural industry integration across 31 provinces (municipalities and autonomous regions) in China.

**the Tobit model.** The efficiency of rural industrial integration measured by the super-efficiency EBM model is all lower bounded by 0, which has certain limitations, while the Tobit model is a statistical method used to deal with the problem of restricted dependent variables, especially suitable for the situation where the dependent variable takes a certain value, which can accurately capture the data characteristics and give a reasonable parameter estimation, avoiding the problem of estimation bias due to the least-squares method [23]. Therefore, the Tobit model was used to analyze the factors influencing the efficiency of rural industrial integration. The specific model is illustrated as shown in Equation (4.1):


$$E_{i}=\alpha +\beta_{i}x_{i}+\varepsilon (i=1,2,...,n)$$ (4.1)

Among them, *E*_*i*_ is the dependent variable, representing the rural industry integration efficiency in China’s eastern, central, and western regions, as calculated separately by the non-expected non-angular super-efficiency EBM model mentioned above, *β*_*i*_ represents the estimated parameters, and *x*_*i*_ denotes the explanatory variables, including: industrial structure, industrial extension, infrastructure, human capital, and consumption level, *ε* is the disturbance term, and *α* is the constant.

### Data sources

The panel data of 31 provinces (municipalities and autonomous regions) in China from 2012 to 2022 are used to measure the efficiency of rural industrial integration in the east, central and west of China, and to regress the factors affecting the efficiency of rural industrial integration, and the data are obtained from: 《China Statistical Yearbook》, 《China Rural Statistical Yearbook》, 《China Leisure Farming Yearbook》, 《China Statistical Yearbook of Population and Employment》, and the National Bureau of Statistics (NBS), and the data are supplemented by linear interpolation method for the missing data of the individual years.

### Construction of the indicator system

Based on the connotation of rural industrial integration, referring to relevant research results, comprehensively considering the relationship between rural economic growth, social benefits, resource conservation and environmental protection, and following the scientific, comprehensive, representative and available data, nine indicators were selected to construct the efficiency of rural industrial integration evaluation index system, including five input indicators, three desired output indicators and one non-desired output indicator, as shown in Table 1. In addition, the indicators were logarithmized to avoid the effect of heteroscedasticity.

**Table 1:** Evaluation index system of the efficiency of rural industrial integration.

Indicator category	Norm	Indicator name	Interpretation of indicators	References
Input	Land input	Crop sown area (hectare)	Area sown or transplanted on all land (cropland or non-cropland) for harvested crops during the calendar year	
	Labor input	Rural practitioners (ten thousand people)	Persons in the rural population aged 16 years or older who are actually participating in productive activities and earning income in kind or money	
	Capital input	Expenditures on agriculture, forestry and water affairs (billion yuan)	Including agricultural expenditure, forestry expenditure, water conservancy expenditure, poverty alleviation expenditure, comprehensive agricultural development expenditure, etc.	[16,17,24]
	Mechanical input	Total power of agricultural machinery (million kilowatts)	Sum of power ratings of all agricultural machinery power	[24,25]
	Fertilizer input	Application of agricultural fertilizers (million tons)	Quantity of fertilizer actually used in agricultural production during the year, including nitrogen, phosphate, potash and compound fertilizer	[24,26]
Expected outputs	Value of agricultural production	Agricultural output per unit area (yuan/hectare)	Agricultural output/cultivated land	
	Farmers’ income	Farmers’ income level (yuan)	Per capita disposable income of rural residents	[16,17,25,26]
		Operating income from leisure tourism (100 million yuan)	Operating income from leisure agriculture and rural tourism	[24,26]
Non-expected outputs	Environmental pollution	Agricultural plastic film coverage (tons/hectare)	Agricultural plastic film use/area sown to crops	[14]

Taking the efficiency of rural industrial integration as the explanatory variable, referring to relevant research results, comprehensively considering the relationship between industrial structure, industrial extension, infrastructure, human capital, and consumption level, and at the same time following the scientific, comprehensive, representative and available nature of the data, one explanatory variable and five explanatory variables are selected to construct the evaluation index system factors of the efficiency of rural industrial integration, as shown in Table 2. In addition, the indicators were logarithmized to avoid the effect of heteroscedasticity.

**Table 2:** Evaluation index system of factors influencing the efficiency of rural industrial integration.

Variable type	Variable name	Variable meaning	Unit	References
Explained variable	Rural industrial integration efficiency	It is calculated by using the non-expectation non-angle super-efficiency EBM model	─	
Explanatory variable	Industrial structure	Value added of tertiary industry/GDP	%	
	Industrial extension	Value added of primary industry/GDP	%	
	Infrastructure	Average year-end cell phone ownership per 100 rural households	Cell	
	Human capital	Educational attainment per capita in rural areas = (sum of the number of rural people with elementary school education multiplied by 6, the number of rural people with lower secondary school education multiplied by 9, the number of rural people with upper secondary school education multiplied by 12, and the number of rural people with specialized education or above multiplied by 16) divided by the number of people aged 6 years and above	Year	[27,28]
	Consumption level	Consumption expenditure per rural inhabitant	Yuan	[29]

## Analysis of empirical results

### Analysis of the results of measuring the efficiency of rural industrial integration efficiency

Using MaxDEA software, the non-expectation non-angle super-efficiency EBM model was applied to measure the efficiency and decomposition terms of China’s rural industrial integration from 2012 to 2022, as shown in Table 3.

**Table 3:** Efficiency of rural industrial integration and its decomposition terms in eastern China, 2012-2022.

			2012	2013	2014	2015	2016	2017	2018	2019	2020	2021	2022	Average	Ranking
East	Combined	Beijing	1.027	1.038	1.058	1.080	1.115	1.204	1.279	1.325	1.257	1.216	1.186	1.162	2
		Tianjin	1.053	1.043	1.050	1.051	1.056	1.175	1.194	1.207	1.230	1.266	1.273	1.145	3
		Hebei	0.000	0.0002	0.002	0.018	0.129	0.352	0.471	0.430	0.391	0.353	0.316	0.224	11
		Liaoning	0.001	0.008	0.040	0.175	0.348	0.508	0.532	0.414	0.353	0.311	0.271	0.269	10
		Shanghai	1.169	1.156	1.160	1.156	1.142	1.152	1.140	1.122	1.105	1.087	1.075	1.133	4
		Jiangsu	1.013	1.010	1.004	1.012	1.013	1.013	1.107	1.106	1.107	1.108	1.108	1.055	5
		Zhejiang	1.002	1.005	0.056	0.245	0.680	1.073	1.058	1.067	1.072	1.078	1.084	0.856	8
		Fujian	1.003	1.014	1.023	1.013	0.558	0.825	0.901	1.012	1.030	1.048	1.042	0.952	7
		Shandong	0.001	0.004	0.018	0.078	0.284	0.555	0.642	0.568	0.529	0.494	0.450	0.329	9
		Guangdong	1.274	1.277	1.289	1.262	1.282	1.225	1.127	1.135	1.142	1.146	1.150	1.210	1
		Hainan	1.004	1.015	1.024	1.045	1.067	1.062	1.059	1.056	1.057	1.062	1.057	1.046	6
		Average	0.777	0.779	0.702	0.740	0.789	0.922	0.955	0.949	0.934	0.924	0.910	─	─
	Pure technical	Beijing	1.047	1.057	1.083	1.107	1.150	1.247	1.295	1.352	1.320	1.264	1.216	1.194	4
		Tianjin	1.187	1.158	1.180	1.231	1.272	1.535	1.583	1.790	1.805	1.835	1.941	1.501	1
		Hebei	0.000	0.0003	0.002	0.019	0.132	0.378	0.554	0.503	0.476	0.439	0.393	0.263	11
		Liaoning	0.002	0.008	0.043	0.180	0.374	0.518	0.533	0.428	0.367	0.324	0.291	0.279	10
		Shanghai	1.261	1.294	1.301	1.291	1.224	1.223	1.256	1.256	1.312	1.350	1.422	1.290	3
		Jiangsu	1.014	1.011	1.007	1.012	1.016	1.014	1.110	1.110	1.111	1.111	1.111	1.057	7
		Zhejiang	1.014	1.017	1.016	1.006	1.010	1.095	1.119	1.132	1.140	1.144	1.147	1.076	5
		Fujian	1.005	1.016	1.025	1.014	1.019	1.019	1.021	1.025	1.033	1.048	1.048	1.025	8
		Shandong	0.001	0.004	0.019	0.084	0.323	0.615	0.670	0.576	0.530	0.495	0.450	0.342	9
		Guangdong	1.402	1.387	1.387	1.359	1.422	1.341	1.206	1.207	1.207	1.204	1.203	1.302	2
		Hainan	1.035	1.044	1.041	1.055	1.067	1.064	1.065	1.068	1.067	1.068	1.065	1.058	6
		Average	0.815	0.818	0.828	0.851	0.910	1.004	1.037	1.041	1.033	1.026	1.026	─	─
	Scale	Beijing	0.981	0.982	0.978	0.975	0.969	0.965	0.987	0.980	0.952	0.962	0.975	0.973	3
		Tianjin	0.888	0.901	0.889	0.854	0.830	0.765	0.754	0.674	0.681	0.690	0.656	0.780	11
		Hebei	0.957	0.963	0.987	0.975	0.976	0.931	0.849	0.856	0.822	0.803	0.804	0.902	8
		Liaoning	0.921	0.926	0.930	0.973	0.930	0.980	0.998	0.967	0.962	0.958	0.933	0.953	4
		Shanghai	0.927	0.893	0.891	0.895	0.933	0.942	0.908	0.894	0.843	0.806	0.756	0.881	9
		Jiangsu	0.999	1.000	0.997	0.999	0.998	0.999	0.997	0.997	0.997	0.998	0.998	0.998	1
		Zhejiang	0.989	0.988	0.055	0.243	0.673	0.980	0.945	0.942	0.940	0.942	0.945	0.786	10
		Fujian	0.998	0.997	0.998	1.000	0.547	0.810	0.882	0.988	0.996	1.000	0.994	0.928	7
		Shandong	0.939	0.930	0.926	0.925	0.881	0.902	0.959	0.987	0.999	0.998	0.999	0.950	5
		Guangdong	0.908	0.921	0.929	0.929	0.902	0.914	0.935	0.941	0.947	0.952	0.956	0.930	6
		Hainan	0.970	0.973	0.983	0.990	1.000	0.998	0.994	0.989	0.990	0.994	0.992	0.988	2
		Average	0.952	0.952	0.869	0.887	0.876	0.926	0.928	0.929	0.921	0.918	0.910	─	─

Notes: *With reference to the standards of the National Bureau of Statistics, China’s 31 provinces (municipalities and autonomous regions) are divided into three regions, namely, the eastern, central and western regions, to explore the changing trends in the efficiency of the integrated development of rural industries in the three regions. The eastern region includes 11 provinces (municipalities and autonomous regions), including Beijing, Tianjin, Hebei, Liaoning, Shanghai, Jiangsu, Zhejiang, Fujian, Shandong, Guangdong and Hainan; the central region includes 8 provinces (municipalities and autonomous regions), including Shanxi, Jilin, Heilongjiang, Anhui, Jiangxi, Henan, Hubei and Hunan; and the western region includes 12 provinces (municipalities and autonomous regions), including Neimenggu, Sichuan, Chongqing, Guangxi, Guizhou, Yunnan, Xizang, Shaanxi, Gansu, Qinghai, Ningxia and Xinjiang.

As shown in Table 3, on the whole, both the combined efficiency of rural industrial integration and pure technical efficiency in the east show a rapid growth trend, and the combined efficiency of rural industrial integration in the east grows from 0.777 in 2012 to 0.910 in 2022, with a growth rate of 17.141%, of which the combined efficiency of rural industrial integration in the east grows the fastest in 2016–2017, growing from 0.789 in 2016 to 0.922 in 2017, an increase of 0.134; the pure technical efficiency of rural industrial integration in the east grew from 0.815 in 2012 to 1.026 in 2022, a growth rate of 25.859%, of which, the pure technical efficiency of rural industrial integration in the east grew the fastest in 2016–2017, from 0.910 in 2016–2017. The main reason is that since the “No. 1 central document” for 2015 first proposed “promoting the integrated development of primary, secondary and tertiary industries in rural areas”, the government and relevant departments have issued a series of policies and documents to support the integrated development of rural industries. So the first results were seen in 2016–2017, while making the integration of rural industries forward. The scale efficiency of rural industrial integration in the east shows a decreasing trend from 0.952 in 2012 to 0.910 in 2022, with a decrease rate of 4.476%. To summarize, at present, pure technical efficiency is the core driving force of the efficiency of rural industrial integration in eastern China, and scale efficiency contributes weakly to the efficiency of rural industrial integration in eastern China, so the deep excavation of the scale efficiency of rural industrial integration in eastern China is the top priority for future work.

Regionally, in terms of the combined efficiency, Shandong, Liaoning, Hebei rural industrial integration combined efficiency ranked 9th, 10th and 11th among the 11 eastern provinces (municipalities and autonomous regions), mainly because these three regions are not balanced in terms of inputs and outputs, inputs are more but outputs are less, and there is the problem of irrational allocation and inefficient utilization of resources. For example, in Hebei Province, there are more inputs in terms of land inputs, machinery inputs and chemical fertilizers, and the farmers’ income in the leisure tourism business is less, resulting in the combined efficiency of rural industrial integration in Hebei Province being in last place. Specifically, because the agricultural product market systems in these three regions are not well developed and there is asymmetry in market information, it is difficult for resource allocation to be optimized through market mechanisms. For example, the lack of effective channels to connect with urban consumer markets makes it challenging to convert high investments into high returns. The fact that Guangdong Province ranks first in terms of combined efficiency of rural industrial integration is attributed to the rational use of resources in Guangdong Province. For example, although Guangdong Province has a high level of labor inputs and capital inputs, it also has a high level of agricultural outputs and operating revenues from the leisure and tourism industry, which creates a recycling network for efficient use. Specifically, the reasons are as follows: Firstly, Guangdong Province enjoys the policy advantages of being a pilot region and a relatively relaxed institutional environment, which provides policy support and a favorable setting for the integration of rural industries. Secondly, Guangdong Province has a high level of economic development, strong technological innovation capabilities, and a mature logistics network and tourism service industry, all of which contribute to the enhancement of the value chain. In terms of pure technical efficiency, the pure technical efficiency of rural industrial integration in Shandong, Liaoning, and Hebei ranked 9th, 10th, and 11th among the 11 eastern provinces (municipalities and autonomous regions), because of the relatively low level of technology, low level of management, and insufficient innovation capacity in these three regions. For example, Liaoning Province’s low machinery inputs made the agricultural output ranked at the bottom of the 11 eastern provinces (municipalities and autonomous regions); the first place in pure technical efficiency of rural industrial integration in Tianjin is attributed to its low labor inputs, capital inputs, machinery inputs, and fertilizer inputs, and the high level of agricultural output and farmers’ incomes. Specifically, due to the small scale of agriculture in Tianjin, the proportion of knowledge and technology-intensive sectors such as leisure agriculture is relatively high. This structure is more reliant on support from advanced technology and management, and the technological development and management level in Tianjin precisely meet these needs. Therefore, although Tianjin invests less, the agricultural output value and farmers’ income levels are comparatively high. In terms of scale efficiency, the scale efficiency of rural industrial integration in Tianjin ranks 11th among 11 eastern provinces (municipalities and autonomous regions) because Tianjin’s pure technical efficiency is too high; the scale efficiency of rural industrial integration in Jiangsu Province ranks first because Jiangsu Province’s pure technical efficiency is low, which suggests that Jiangsu Province has a high potential for development and development of rural industrial integration.

As shown in Table 4, on the whole, the combined efficiency, pure technical efficiency and scale efficiency of rural industrial integration in central China show a growing trend, and the combined efficiency of rural industrial integration in central China grew from 1.004 in 2012 to 1.031 in 2022, with a growth rate of 2.689%; The pure technical efficiency of rural industrial integration in central China grew from 1.025 in 2012 to 1.046 in 2022, a growth rate of 2.048%; The scale efficiency of rural industrial integration in central China decreased from 0.979 in 2012 to 0.986 in 2022, with a growth rate of 0.664%. To summarize, at present, combined efficiency is the core driving force of the efficiency of rural industrial integration in central China, and scale efficiency contributes weakly to the efficiency of rural industrial integration in central China, so the deep excavation of scale efficiency of rural industrial integration in central China is the top priority for future work.

**Table 4:** Efficiency of rural industrial integration and its decomposition terms in central China, 2012-2022.

			2012	2013	2014	2015	2016	2017	2018	2019	2020	2021	2022	Average	Ranking
Central	Combined	Shanxi	1.029	1.022	1.029	1.035	1.039	1.034	1.024	1.019	1.016	1.012	1.010	1.024	5
		Jilin	1.033	1.033	1.033	1.030	1.026	1.023	1.020	1.019	1.018	1.022	1.019	1.025	4
		Heilongjiang	0.897	0.907	0.915	0.900	1.001	1.003	1.002	1.001	1.001	1.003	1.004	0.967	8
		Anhui	1.001	1.014	1.026	1.023	1.026	1.028	1.031	1.038	1.044	1.052	1.062	1.031	3
		Jiangxi	0.969	1.019	1.021	1.025	1.018	1.018	1.036	1.048	1.061	1.069	1.085	1.034	2
		Henan	1.063	1.079	1.068	1.074	1.071	1.074	1.065	1.060	1.057	1.051	1.047	1.064	1
		Hubei	1.035	1.027	1.025	1.017	1.016	1.015	1.011	1.009	1.003	1.003	1.004	1.015	6
		Hunan	1.007	1.010	1.001	1.008	1.007	1.010	1.019	1.016	1.015	1.016	1.019	1.012	7
		Average	1.004	1.014	1.015	1.014	1.026	1.026	1.026	1.026	1.027	1.029	1.031	─	─
	Pure technical	Shanxi	1.038	1.028	1.048	1.056	1.091	1.093	1.092	1.093	1.094	1.100	1.099	1.076	1
		Jilin	1.041	1.043	1.043	1.036	1.029	1.029	1.036	1.038	1.039	1.039	1.037	1.037	4
		Heilongjiang	1.001	1.000	1.001	1.001	1.002	1.004	1.003	1.001	1.001	1.003	1.004	1.002	8
		Anhui	1.003	1.015	1.028	1.024	1.026	1.029	1.031	1.039	1.044	1.052	1.068	1.033	5
		Jiangxi	1.003	1.020	1.022	1.026	1.022	1.024	1.039	1.050	1.063	1.072	1.086	1.039	3
		Henan	1.071	1.083	1.075	1.085	1.079	1.078	1.066	1.063	1.059	1.055	1.050	1.069	2
		Hubei	1.037	1.031	1.026	1.019	1.019	1.018	1.012	1.010	1.005	1.004	1.004	1.017	6
		Hunan	1.008	1.010	1.004	1.010	1.010	1.012	1.024	1.018	1.016	1.019	1.022	1.014	7
		Average	1.025	1.029	1.031	1.032	1.035	1.036	1.038	1.039	1.040	1.043	1.046	─	─
	Scale	Shanxi	0.992	0.995	0.982	0.980	0.953	0.946	0.938	0.932	0.928	0.920	0.919	0.953	8
		Jilin	0.992	0.990	0.990	0.994	0.997	0.994	0.984	0.982	0.980	0.984	0.983	0.988	6
		Heilongjiang	0.896	0.907	0.915	0.899	0.999	0.999	0.999	0.999	1.000	1.000	1.000	0.965	7
		Anhui	0.999	0.998	0.998	1.000	1.000	0.999	1.000	0.999	1.000	1.000	0.994	0.999	1
		Jiangxi	0.967	0.999	0.999	0.999	0.996	0.995	0.997	0.998	0.998	0.998	0.998	0.995	5
		Henan	0.992	0.996	0.993	0.990	0.992	0.996	0.999	0.998	0.998	0.997	0.996	0.995	4
		Hubei	0.998	0.996	0.999	0.997	0.997	0.997	0.999	0.999	0.998	0.999	1.000	0.998	2
		Hunan	0.999	1.000	0.997	0.997	0.997	0.997	0.996	0.999	0.999	0.997	0.997	0.998	3
		Average	0.979	0.985	0.984	0.982	0.991	0.990	0.989	0.988	0.988	0.987	0.986	─	─

Regionally, in terms of the combined efficiency, Hubei, Hunan and Heilongjiang ranked 6th, 7th and 8th among the 8 central provinces (municipalities and autonomous regions) in terms of combined efficiency of rural industrial integration, mainly because of the imbalance of inputs and outputs in these three regions, with more inputs but fewer outputs. For example, more land inputs in Heilongjiang and small agricultural outputs, which led to the last place of the combined efficiency of rural industrial integration in Heilongjiang Province. Specifically, the reason is that Heilongjiang Province relies on land-intensive production, lacking support from high value-added segments, resulting in a high input of land but a small agricultural output. Henan Province ranks first in terms of combined efficiency of rural industrial integration, mainly thanks to the rational use of resources in Henan Province. For example, although Henan Province has more land inputs, labor inputs, capital inputs, machinery inputs, and fertilizer inputs, it also has a high level of agricultural output and farmers’ incomes, and achieves efficient use of resources. Specifically, the reason is that Henan Province is a significant food industry base in the country, capable of transforming abundant agricultural products locally into high value-added industrial goods, effectively extending the industrial chain and enhancing the value chain. In terms of pure technical efficiency, the pure technical efficiency of rural industrial integration in Hubei, Hunan, and Heilongjiang ranked 6th, 7th, and 8th among the 8 central provinces (municipalities and autonomous regions), because of the relatively low level of technology, low level of management, and insufficient innovation capacity in these three regions, for example, the pure technical efficiency of rural industrial integration in Hubei ranked the third from the bottom among the 8 provinces (municipalities and autonomous regions), because of the relatively low investment in machinery and the technological inputs are not in place; The pure technical efficiency of rural industrial integration in Shanxi Province is in the first place, mainly because of the balanced inputs and outputs, with less land inputs, machinery inputs, and fertilizer inputs, while the level of agricultural outputs and farmers’ incomes are also less, and there is no waste of resources. In terms of scale efficiency, the scale efficiency of rural industrial integration in Shanxi Province ranks 8th among the 8 provinces (municipalities and autonomous regions) in central China, mainly because of the excessive pure technical efficiency in Shanxi Province; The scale efficiency of rural industrial integration in Anhui Province is in the first place, thanks to the high combined efficiency and low pure technical efficiency of Anhui Province, which indicates that Anhui Province has a high potential for the development of rural industrial integration.

As shown in Table 5, on the whole, the combined efficiency and scale efficiency of rural industrial integration in the west show a decreasing trend, and the combined efficiency of rural industrial integration in the west decreases from 0.995 in 2012 to 0.968 in 2022, with a decrease rate of 2.771%; The scale efficiency of rural industrial integration in western China decreases from 0.996 in 2012 to 0.963 in 2022, with a decline rate of 3.256%; The pure technical efficiency of rural industrial integration in the west shows an increasing trend from 1.000 in 2012 to 1.005 in 2022, with a growth rate of 0.509%. To summarize, at present, pure technical efficiency is the core driving force of the efficiency of rural industrial integration in western China, and scale efficiency contributes weakly to the efficiency of rural industrial integration in western China, so the deep excavation of the scale efficiency of rural industrial integration in western China is the top priority for future work.

**Table 5:** Efficiency of rural industrial integration and its decomposition terms in western China, 2012-2022.

			2012	2013	2014	2015	2016	2017	2018	2019	2020	2021	2022	Average	Ranking
West	Combined	Neimenggu	1.005	1.006	1.004	1.003	1.006	1.005	1.006	1.008	1.011	1.013	1.027	1.009	7
		Guangxi	1.008	1.013	1.017	1.016	1.015	1.012	1.016	1.026	1.034	1.065	1.101	1.029	2
		Chongqing	1.010	1.010	1.012	1.014	1.017	1.021	1.027	1.033	1.037	1.032	1.035	1.023	3
		Sichuan	1.014	1.013	1.011	1.013	1.014	1.014	1.015	1.010	1.003	0.917	0.897	0.993	9
		Guizhou	0.868	0.863	0.857	0.870	0.870	0.865	0.869	0.860	0.856	0.851	0.829	0.860	12
		Yunnan	0.919	0.922	0.881	0.884	0.905	0.894	0.906	0.903	0.883	0.870	0.858	0.893	11
		Xizang	1.006	1.006	1.005	1.006	1.006	1.008	1.009	1.011	1.014	1.018	1.019	1.010	6
		Shaanxi	1.018	1.018	1.012	1.011	1.017	1.015	1.017	1.013	1.016	1.017	1.017	1.016	4
		Gansu	1.005	1.005	1.004	1.006	1.019	1.013	1.010	1.008	1.006	0.884	0.832	0.981	10
		Qinghai	1.010	1.014	1.015	1.012	1.010	1.012	1.011	1.010	1.008	1.005	0.959	1.006	8
		Ningxia	1.010	1.010	1.011	1.014	1.013	1.009	1.014	1.017	1.012	1.008	1.006	1.011	5
		Xinjiang	1.070	1.074	1.101	1.099	1.082	1.093	1.084	1.076	1.056	1.042	1.032	1.074	1
		Average	0.995	0.996	0.994	0.996	0.998	0.997	0.999	0.998	0.995	0.977	0.968	─	─
	Pure technical	Neimenggu	1.007	1.007	1.007	1.007	1.007	1.008	1.008	1.009	1.015	1.014	1.033	1.011	9
		Guangxi	1.008	1.013	1.018	1.018	1.016	1.014	1.021	1.030	1.038	1.070	1.107	1.032	2
		Chongqing	1.012	1.014	1.016	1.020	1.023	1.028	1.031	1.035	1.038	1.033	1.036	1.026	3
		Sichuan	1.019	1.017	1.018	1.022	1.025	1.027	1.027	1.023	1.016	1.004	1.004	1.018	5
		Guizhou	0.889	0.889	0.881	0.885	0.886	0.889	0.890	0.874	0.865	0.857	0.829	0.876	12
		Yunnan	0.927	0.925	0.899	0.896	0.909	0.903	0.910	0.903	0.910	0.900	0.893	0.907	11
		Xizang	1.006	1.006	1.007	1.007	1.007	1.009	1.010	1.011	1.015	1.019	1.019	1.010	10
		Shaanxi	1.019	1.020	1.012	1.012	1.021	1.020	1.026	1.022	1.030	1.031	1.031	1.022	4
		Gansu	1.011	1.010	1.008	1.013	1.052	1.030	1.021	1.018	1.013	1.007	1.005	1.017	6
		Qinghai	1.012	1.016	1.016	1.014	1.010	1.014	1.013	1.012	1.012	1.012	1.011	1.013	8
		Ningxia	1.010	1.010	1.011	1.014	1.015	1.012	1.017	1.022	1.018	1.017	1.022	1.015	7
		Xinjiang	1.075	1.078	1.107	1.104	1.082	1.093	1.092	1.092	1.086	1.076	1.066	1.086	1
		Average	1.000	1.000	1.000	1.001	1.004	1.004	1.006	1.004	1.005	1.003	1.005	─	─
	Scale	Neimenggu	0.998	0.999	0.997	0.996	0.998	0.997	0.998	0.999	0.997	0.999	0.994	0.997	3
		Guangxi	1.000	1.000	0.999	0.998	0.999	0.998	0.996	0.997	0.997	0.995	0.995	0.998	2
		Chongqing	0.997	0.996	0.995	0.995	0.995	0.994	0.996	0.999	0.999	0.999	0.999	0.997	4
		Sichuan	0.996	0.996	0.993	0.991	0.990	0.988	0.989	0.987	0.987	0.913	0.894	0.975	11
		Guizhou	0.977	0.971	0.973	0.983	0.982	0.973	0.977	0.984	0.989	0.994	1.000	0.982	10
		Yunnan	0.991	0.996	0.980	0.986	0.996	0.991	0.996	1.000	0.971	0.966	0.961	0.985	9
		Xizang	1.000	0.999	0.999	1.000	1.000	0.999	0.999	1.000	1.000	1.000	1.000	0.999	1
		Shaanxi	0.999	0.998	1.000	0.999	0.995	0.995	0.992	0.991	0.986	0.986	0.987	0.994	6
		Gansu	0.994	0.995	0.996	0.994	0.968	0.983	0.989	0.990	0.993	0.878	0.827	0.964	12
		Qinghai	0.999	0.998	0.999	0.999	1.000	0.998	0.998	0.998	0.997	0.993	0.949	0.993	7
		Ningxia	1.000	1.000	1.000	1.000	0.998	0.997	0.997	0.995	0.994	0.991	0.984	0.996	5
		Xinjiang	0.996	0.996	0.994	0.996	1.000	1.000	0.993	0.985	0.972	0.968	0.968	0.988	8
		Average	0.996	0.995	0.994	0.995	0.993	0.993	0.993	0.994	0.990	0.974	0.963	─	─

Regionally, in terms of the combined efficiency, Gansu, Yunnan and Guizhou rural industrial integration combined efficiency ranked 10th, 11th and 12th of the 12 western provinces (municipalities, autonomous regions), mainly because of the imbalance of inputs and outputs in these three regions, there is irrational allocation of resources, the use of inefficient. For example, Yunnan Province has more land inputs, labor inputs, and fertilizer inputs, and less operating income from leisure tourism, resulting in the second-lowest combined efficiency of rural industrial integration in Yunnan Province. Specifically, the reasons are as follows: Firstly, the ecological fragility of the Yungui Plateau necessitates the implementation of strict ecological protection policies, which limits the development space for extensive leisure tourism. Secondly, the industrial chains in Gansu Province, Yunnan Province, and Guizhou Province are short and lack deep processing links, leading to leisure agriculture often remaining at a lower level of sightseeing, with unique resources not integrated into the industrial chain to form competitive advantages. Xinjiang ranks first in terms of combined efficiency of rural industrial integration, because of lower labor inputs and higher levels of farmers’ income. Specifically, the reason is that the level of agricultural mechanization in Xinjiang is high. For instance, in 2020, Xinjiang will promote comprehensive agricultural mechanization through 21 supporting measures across seven key areas, effectively enhancing the mechanization level in major sectors such as crop cultivation, animal husbandry, forestry and fruit cultivation, aquaculture, and the agricultural product processing industry. By 2021, the focus has shifted from full mechanization of major crops to a comprehensive, high-end, and intelligent development, initiating a new round of iterative upgrades. In terms of pure technical efficiency, the pure technical efficiency of rural industrial integration in Xizang, Yunnan, and Guizhou ranked 10th, 11th, and 12th among the 12 western provinces (municipalities and autonomous regions), mainly because of the relatively low level of technology, low level of management, and insufficient innovation capacity in these three regions. For example, less mechanical inputs in the Xizang resulted in less agricultural output and less operating income from leisure tourism; Xinjiang ranks first in terms of pure technical efficiency of rural industrial integration, thanks to its lower labor inputs and higher level of farmers’ income. In terms of scale efficiency, the scale efficiency of rural industrial integration in Gansu Province ranks at the bottom of the 12 western provinces (municipalities and autonomous regions), mainly because of the low combined efficiency of rural industrial integration in Gansu Province; the scale efficiency of rural industrial integration in the Xizang is in the first place because the pure technical efficiency of the Xizang Autonomous Region is low, which indicates that there is a large potential for the development of rural industrial integration in Xizang.

### Regional differences in the efficiency of rural industrial integration

Using SPSS software, the Dagum Gini coefficient was applied to measure and decompose the efficiency of rural industrial integration in China from 2012 to 2022 in order to reveal the degree of spatial differentiation, as shown in Tables 6 and 7.

**Table 6:** Dagum Gini coefficients for intra- and inter-regional rural industrial integration efficiency in three major regions of China, 2012-2022.

Year	Intra-regional Dagum Gini coefficient			Inter-regional Dagum Gini coefficient		
	East	Central	West	East-Central	East-West	Central-West
2012	0.303	0.025	0.022	0.188	0.187	0.026
2013	0.301	0.021	0.023	0.184	0.186	0.024
2014	0.378	0.019	0.028	0.240	0.245	0.027
2015	0.333	0.022	0.027	0.215	0.218	0.027
2016	0.270	0.010	0.024	0.185	0.188	0.020
2017	0.174	0.010	0.026	0.122	0.128	0.021
2018	0.150	0.009	0.025	0.107	0.113	0.020
2019	0.168	0.010	0.026	0.116	0.124	0.021
2020	0.176	0.012	0.027	0.121	0.130	0.022
2021	0.185	0.013	0.039	0.127	0.142	0.033
2022	0.195	0.015	0.049	0.132	0.151	0.042
Average	0.239	0.015	0.029	0.158	0.165	0.026

**Table 7:** Contribution of sources of spatial variation in the efficiency of rural industrial integration in China, 2012-2022.

		2012	2013	2014	2015	2016	2017	2018	2019	2020	2021	2022	Average
Contribution rate (%)	Intra-regional	29.906	29.954	27.966	28.401	27.825	29.367	29.377	29.720	29.582	29.753	30.172	29.275
	Inter-regional	44.560	46.310	50.877	48.451	47.476	26.658	19.911	19.953	23.292	23.239	24.999	34.157
	Hyper-variable density	25.534	23.737	21.157	23.147	24.700	43.976	50.711	50.327	47.126	47.009	44.829	36.568

As shown in Table 6, the intra-regional Dagum Gini coefficients of rural industrial integration efficiency in eastern and central China generally show a decreasing trend, indicating that the overall gap in rural industrial integration efficiency within the eastern and central regions of China has narrowed and the spatial imbalance type has weakened; The intra-regional Dagum Gini coefficient of the efficiency of rural industrial integration in western China is generally on the rise, indicating that the overall gap in the efficiency of rural industrial integration within the region of western China has widened and the spatial imbalance type has intensified. Specifically, the eastern Dagum Gini coefficient decreased from 0.303 in 2012 to 0.195 in 2022, the reason is that some coastal core provinces in eastern China, such as Shanghai, Zhejiang, and Guangdong, have been the first to develop integrated rural tourism and e-commerce logistics, promoting the integrated development of rural industries. As costs rise and competition intensifies, the industrial models, advanced technologies, and management expertise from these areas are beginning to transfer to relatively slower-developing regions like Hebei and Shandong in the eastern part of the country, thereby driving development in Hebei and Shandong. Consequently, the overall gap in rural industrial integration efficiency within the eastern region of China is narrowing, and spatial inequality is diminishing. The central Dagum Gini coefficient decreased from 0.025 in 2012 to 0.015 in 2022, the reason is that the central region serves as a primary base for the industrial transfer from the eastern region. With the relocation of agricultural processing and manufacturing industries to areas such as Henan, Anhui, Jiangxi, Hunan, and Hubei, the impact is extensive and the benefits are significant. This has led to the widespread promotion of the ‘company-cooperative-farmer’ model, enhancing the efficiency of industry integration in rural areas. Consequently, the overall gap in the efficiency of rural industrial integration within China’s central region has been narrowing, and the spatial imbalance is diminishing. The Gini coefficient in Western Dagum increased from 0.022 in 2012 to 0.049 in 2022. This rise is attributed to the rapid development of national growth poles such as the Chengdu-Chongqing economic circle and the Guanzhong Plain urban agglomeration, which have attracted a significant amount of high-quality resources within the region, including highly skilled talent and ample funding. These factors have laid a solid foundation for the integration of rural industries in these areas. However, rural regions far from these growth poles, such as remote areas in Qinghai, Xizang, and Guizhou, are struggling to benefit from effective spillover effects, resulting in a widening gap in the overall efficiency of rural industrial integration in the western region.

In terms of the intra-regional Dagum Gini coefficient, the largest degree of divergence is in the eastern region, with the average value of the Dagum Gini coefficient for 2012–2022 being 0.239, indicating that the gap in the efficiency of rural industrial integration among regions in eastern China is the largest, the reason lies in the fact that the coastal plain areas in the eastern region, such as the Yangtze River Delta and the Pearl River Delta, possess superior natural conditions and a solid development foundation. Additionally, they have a high level of economic development and substantial market demand. In contrast, areas like southwestern Zhejiang and southern Shandong, which are mountainous and hilly, face relatively constrained natural conditions, lower levels of economic development, and reduced market demand. Therefore, the disparities in rural industrial integration efficiency among various regions in eastern China are primarily attributed to natural conditions, economic development, and market demand. The smallest degree of divergence is in the central region, with the average value of the Dagum Gini coefficient for 2012–2022 being 0.015, indicating that the gap in the efficiency of rural industrial integration among regions in central China is the smallest, the reason is that the central region bears the heavy responsibility of ensuring national food security, and the national support policies for agricultural and rural development are extensive, which plays a certain role in mitigating the excessive differentiation of rural industrial integration within the region. In terms of the trend of change, the overall Dagum Gini coefficient in the east showed an upward and then fluctuating downward trend of change, reaching the highest value (0.333) in 2015; the Dagum Gini coefficient in the center showed a fluctuating downward trend; and the Dagum Gini coefficient in the west showed a fluctuating upward trend.

As shown in Table 6, the Dagum Gini coefficient of the efficiency of rural industrial integration in eastern and central China, and eastern and western China shows an overall downward trend, indicating that the gap in the efficiency of rural industrial integration between eastern and central China, and eastern and western China has narrowed, which contributes to the common development of rural industrial integration in eastern and central China, and eastern and western China; The Dagum Gini coefficient of the efficiency of rural industrial integration in central and western China is generally on the rise, indicating that the gap in the efficiency of rural industrial integration between central and western China has intensified, which is not conducive to the joint development of rural industrial integration in central and western China. Specifically, the eastern and central Dagum Gini coefficient decreases from 0.188 in 2012 to 0.132 in 2022, the reason is that major cities such as Beijing, Shanghai, Guangzhou, and Shenzhen have a high demand for high-quality green agricultural products, while the eastern and central regions are adjacent. This drives the ecological agricultural premium in the central region and helps bridge the gap between the two areas. The eastern and western Dagum Gini coefficient decreases from 0.187 in 2012 to 0.151 in 2022, the reason is that the central government’s fiscal transfer payments are inclined towards the western regions. For instance, the special funds for rural revitalization are prioritized for investment in key grain-producing areas and ecological protection zones in the west. This has significantly improved the efficiency of rural industrial integration in the western regions, thereby narrowing the gap with the efficiency of rural industrial integration in the eastern regions. And the central and western Dagum Gini coefficient increases from 0.026 in 2012 to 0.042 in 2022, the reason is that the western region is relatively underdeveloped, resulting in a significant outflow of young and middle-aged populations, which is not conducive to the integration of rural industries. In contrast, the central region serves as the main hub for industrial relocation, thus attracting a large number of laborers to return, which is beneficial for the integration of rural industries.

On the inter-regional Dagum Gini coefficient, the spatial divergence between the east and the west is the largest in 2012–2022, and the mean value of the inter-regional Dagum Gini coefficient is 0.165, indicating that the gap in the efficiency of rural industrial integration is the largest between the eastern and western regions of China; The central and western regions have the smallest spatial divergence, and the mean value of the Dagum Gini coefficient is 0.026, indicating that the gap in the efficiency of rural industrial integration is the smallest between the central and western regions of China. In terms of trends, the trends of the Dagum Gini coefficients in eastern and central China, and in eastern and western China, were broadly consistent, all showing an upward and then a downward trend, and all reaching their maximum values in 2014 (0.240 and 0.245, respectively); and the trends of the Dagum Gini coefficients in central and western China as a whole showed a fluctuating upward trend.

As shown in Table 7, on the whole, China’s rural industrial integration efficiency has the highest hyper-variable density contribution rate, with a mean hyper-variable density contribution rate of 36.568% from 2012 to 2022; The intra-regional contribution was the lowest, with a mean intra-regional variance contribution of 29.275% over the sample period. In terms of the magnitude of change, the largest change was in the inter-regional contribution rate, which fell by 19.561%; the larger change was in the hyper-variable density contribution rate, which rose by 19.295%; and the smallest change was in the intra-regional contribution rate, which rose by 0.266%.

In terms of the trend of change, the contribution of intra-regional variation over the sample period showed a slightly increasing trend from 29.906% in 2012 to 30.172% in 2022, while the contribution of hyper-variable density showed a significant increasing trend from 25.534% in 2012 to 44.829% in 2022; The contribution rate of inter-regional differences shows a sharp downward trend from 44.560% in 2012 to 24.999% in 2022, which shows that the contribution rates of the current sources of spatial differentiation in the efficiency of rural industrial integration in China are, in order, hyper-variable density > inter-regional differences > intra-regional differences.

### Evolution of the dynamic distribution of the efficiency of rural industrial integration

**Fig 1:**
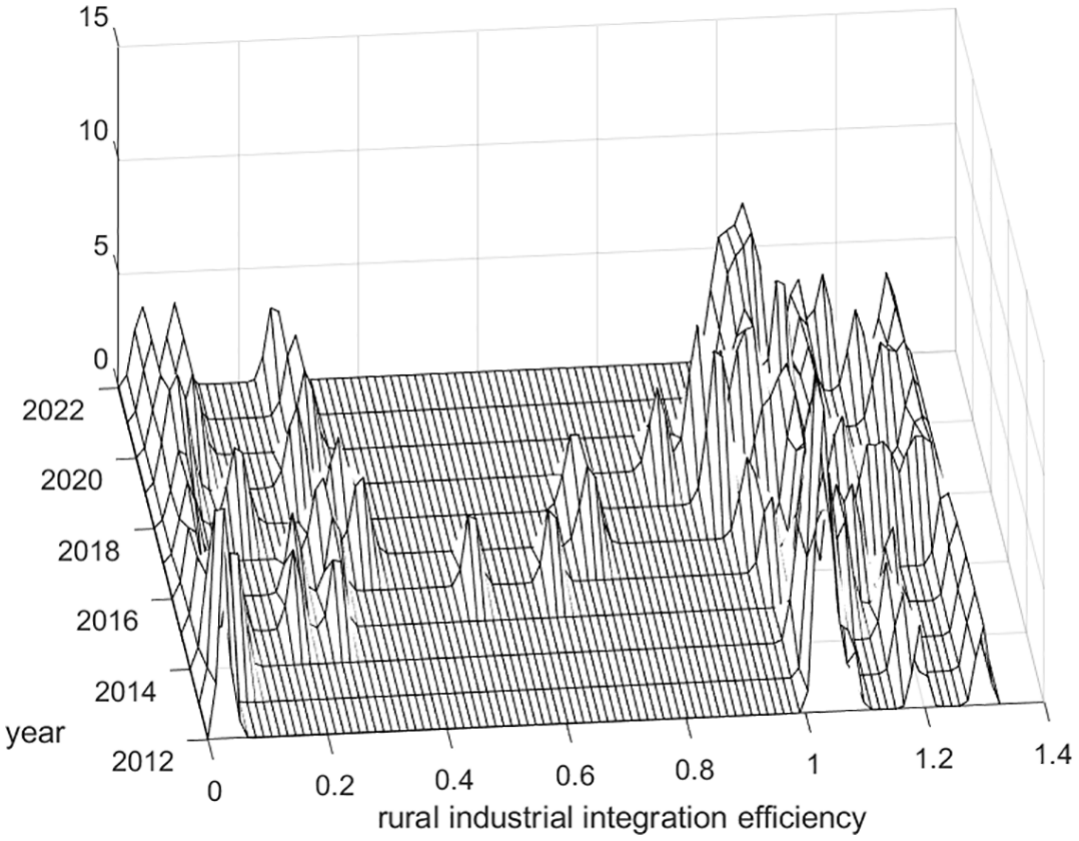
Dynamic distribution of kernel density of rural industrial integration efficiency in eastern China.

**Fig 2:**
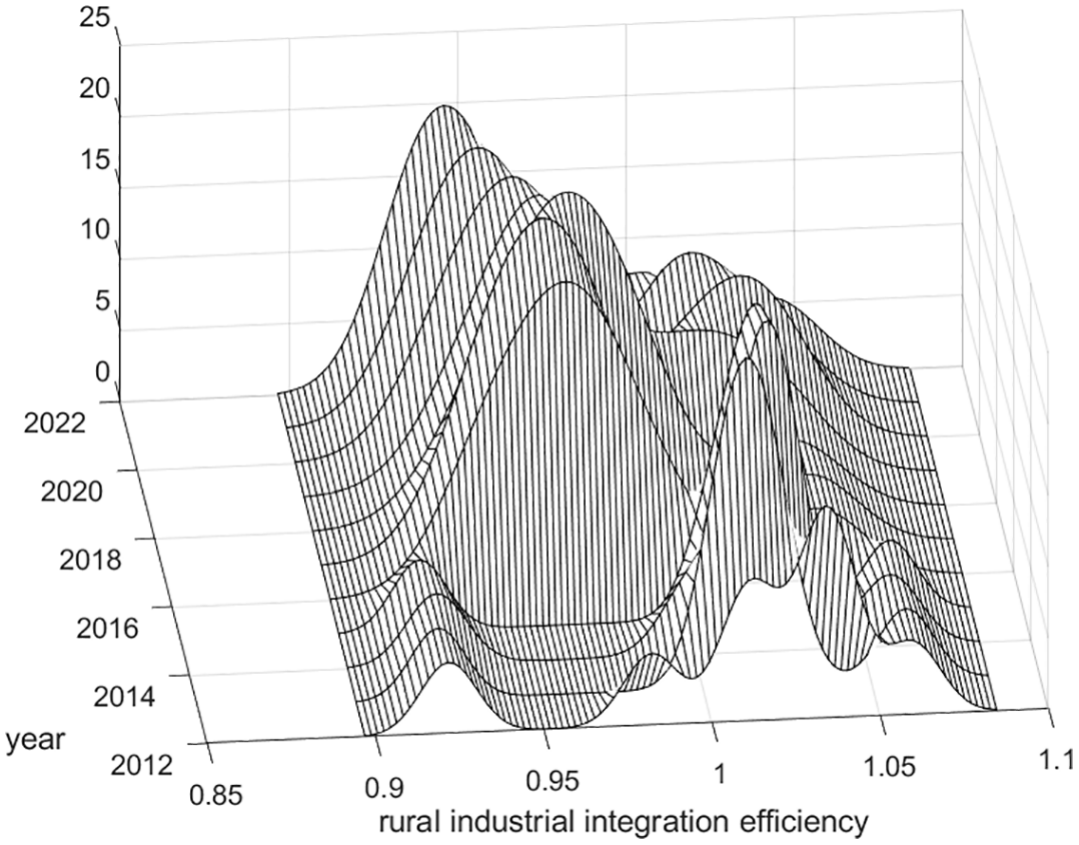
Dynamic distribution of kernel density of rural industrial integration efficiency in central China.

**Fig 3:**
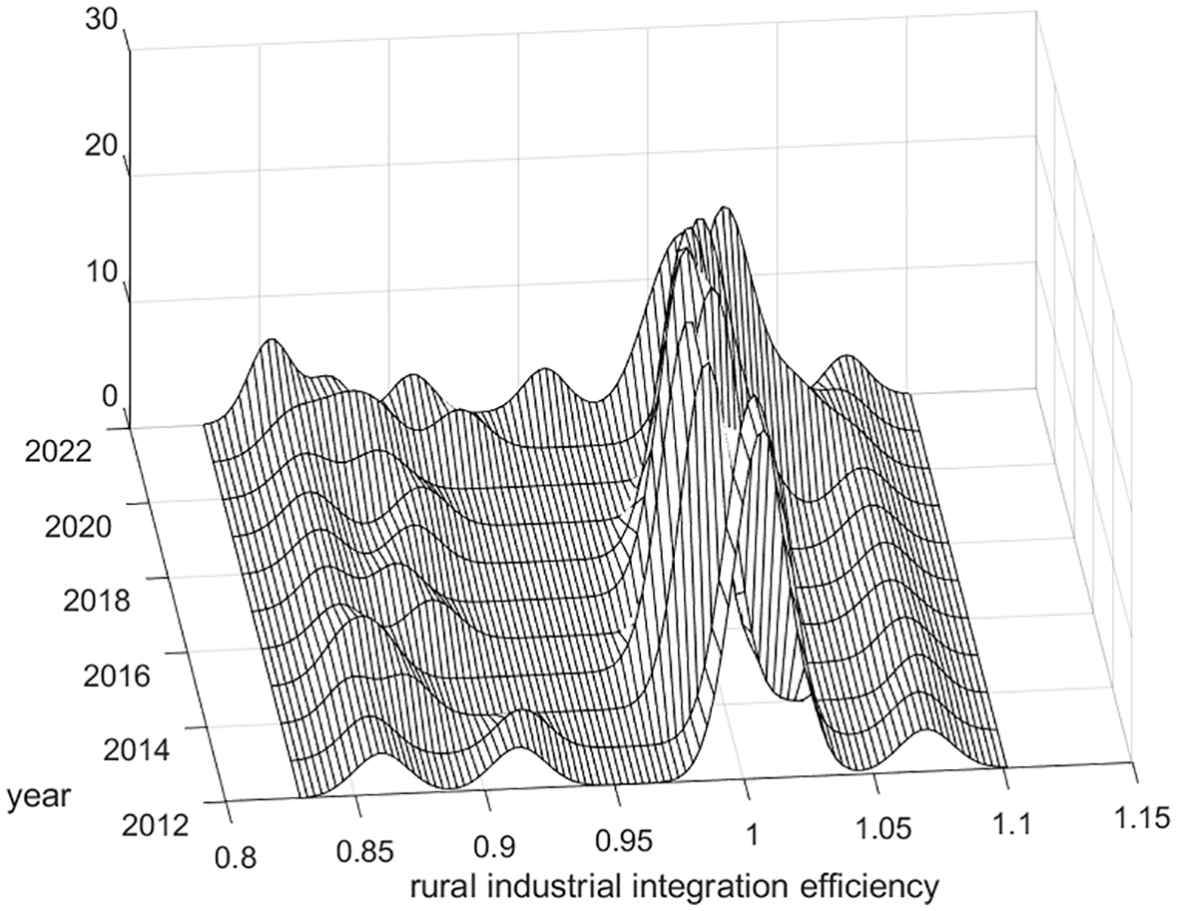
Dynamic distribution of kernel density of rural industrial integration efficiency in western China.

Using MATLAB software, the kernel density estimation method was applied to analyze the efficiency of rural industrial integration in China from 2012 to 2022 in order to reveal its dynamic distribution characteristics, as shown in Figs 1–3.

As shown in Fig 1, the distribution curve of the three-dimensional kernel density in eastern China from 2012 to 2022 shows a multi-peak trend, and the peaks are mostly concentrated between 0–0.4 and 1–1.4, which indicates that the efficiency of rural industrial integration in eastern China is in a bipolar pattern and is seriously differentiated; the right peak is higher than the left peak as a whole, which indicates that the efficiency of rural industrial integration among the regions in eastern China is trending towards the high value of the agglomeration. In addition to the peaks clustered at both ends of the three-dimensional kernel density distribution curve in eastern China in 2016, 2017, and 2018, there were two small peaks between 0.4 and 0.8, a small peak between 0.6 and 0.8, and a small peak between 0.6 and 0.8, respectively, indicating that the efficiency of rural industrial integration in some provinces (municipalities and autonomous regions) in the eastern region is clustered between 0.4 and 0.8, 0.6 and 0.8, but the agglomeration is weak.

As shown in Fig 2, the distribution curve of the three-dimensional kernel density in central China shows an overall multi-peak trend from 2012 to 2022, indicating that there is a trend of differentiation in the efficiency of rural industrial integration in central China. In 2013–2015, the kernel density curve showed the phenomenon of “three peaks”, with a large peak and two small peaks, a large peak in the middle and two small peaks on both sides, and a weak ductility of one main peak and a strong ductility of the two measured peaks, indicating that the efficiency of rural industrial integration of the provinces (municipalities and autonomous regions) of central China has serious clustering phenomena between 1–1.05, and there are clustering phenomena between 0.9–0.95 and 1.05–1.1. In 2016–2022, the distribution curve of three-dimensional kernel density in central China shows an overall trend of “two peaks”, with the main peak on the left and the secondary peak on the right, and the ductility of the main and secondary peaks shrinks, indicating that although there is still a trend of agglomeration in the efficiency of rural industrial integration in central China, the gap of the efficiency of rural industrial integration between central China’s regions has been narrowing, which contributes to the common development of rural industrial integration in central China. It contributes to the common development of rural industrial integration in the central region.

As shown in Fig 3, the distribution curve of the three-dimensional kernel density in western China shows an overall multi-peak trend from 2012 to 2022, indicating that there is a trend of differentiation in the efficiency of rural industrial integration among the western regions of China. The main peak is in the middle, mainly clustered around 1, and the measured peaks are on both sides, mainly clustered between 0.8–0.95 and 1.05–1.1, and the main peaks are weakly ductile, while the measured peaks are strongly ductile, indicating that the efficiency of the integration of rural industries in all the provinces (municipalities and autonomous regions) in western China is mostly concentrated around 1. In addition, the main peak ductility narrows from 2012 to 2022, suggesting that the gap in the efficiency of rural industrial integration is narrowing across provinces (municipalities and autonomous regions) in western China.

### Analysis of factors affecting the efficiency of rural industrial integration

**Regression results and analysis.** Using SPSS software, the Tobit model was applied to analyze the influencing factors of the efficiency of rural industrial integration in the east, central and west of China from 2012 to 2022, as shown in Table 8. Among them, the explanatory variables refer to the efficiency of rural industrial integration in the east, middle and west of China as measured by the non-expected non-angle super-efficiency EBM model above, respectively.

**Table 8:** Regression results of Tobit model in eastern, central and western China.

Variable	East	Central	West
lnIS	7.766** (5.077)	0.035 (0.898)	0.021 (0.272)
lnIE	0.152 (0.689)	−0.045** (−5.204)	−0.075** (−3.943)
lnINF	2.555* (2.076)	0.091* (2.413)	−0.190** (−5.506)
lnHC	−15.429** (−4.425)	−0.163* (−1.969)	0.101 (1.951)
lnCL	1.524* (2.461)	−0.028 (−1.451)	0.050* (2.226)
_Cons	−27.723** (−4.505)	0.091 (0.457)	0.481 (1.677)
Log(Sigma)	0.282** (4.390)	−3.600** (−47.764)	−2.900** (−47.125)
N	121	88	132
Likelihood ratio test	X^2^ (5)=74.863,p = 0.000	X^2^ (5)=36.944,p = 0.000	X^2^ (5)=41.987,p = 0.000

Note: **, * indicate significance at the 5% and 10% levels, respectively, with z-values in parentheses. Same as below.

The industrial structure in the explanatory variables is denoted by IS, which refers to the ratio of value added of tertiary industry to the GDP; Industrial extension is denoted by IE and refers to the ratio of value added of primary production to the GDP; Infrastructure is denoted by INF and refers to the average year-end cell phone ownership per 100 rural households; Human capital is denoted by HC and refers to educational attainment per capita in rural areas; Consumption level is denoted by CL and refers to consumption expenditure per rural inhabitant.

Regionally, in the eastern region, firstly, the industrial structure, infrastructure and consumption level show a significant positive relationship on the efficiency of rural industrial integration in eastern China. Among them, the industrial structure has a significant positive impact on the efficiency of rural industrial integration in eastern China, because the current industrial structure of the eastern region tends to mature, especially the rapid development of the tertiary industry has become a strong driving force for the efficiency of rural industrial integration in the eastern region, with the increasing ratio of the added value of the tertiary industry to the GDP, the deep development of rural tourism and rural e-commerce in the eastern region, which will in turn As the ratio of tertiary industry value added to GDP continues to increase, rural tourism and rural e-commerce in the eastern region will develop in depth, thus promoting the improvement of the efficiency of rural industrial integration in eastern China; Infrastructure has a significant positive impact on the efficiency of rural industrial integration in eastern China, because the improvement of rural residents’ information and communication capabilities are beneficial to their access to more market information, agricultural technology and management experience, thus increasing agricultural productivity and farmers’ incomes; Consumption level has a significant positive impact on the efficiency of rural industrial integration in eastern China, because increased consumption of rural residents can not only improve the purchasing power of rural residents, but also stimulate domestic demand and activate the development of rural industrial integration, in addition, it can promote the transformation and upgrading of rural industries, improve the added value and market competitiveness of agricultural products, and thus enhance the efficiency of rural industrial integration in eastern China. Secondly, human capital has a significant negative impact on the efficiency of rural industrial integration in eastern China, because although there are more highly educated talents in the rural areas of eastern China, they are attracted to move to the cities because of their more open-mindedness and high-income environment in the cities, which leads to a lack of relevant professional knowledge and technical support talents in the process of rural industrial integration in the eastern region, and reduces the efficiency of rural industrial integration in the eastern region of China. Thirdly, industrial extension has no effect on the efficiency of rural industrial integration in eastern China, because eastern China is economically developed and focuses more on the development of secondary and tertiary industries, so the ratio of value added of the primary industry to the regional GDP does not directly contribute to the efficiency of rural industrial integration in eastern China.

In the central region, firstly, infrastructure shows a significant positive impact relationship on the efficiency of rural industrial integration in central China, because the application and popularization of information technology means are beneficial to the distribution of agricultural products and improve the efficiency of rural industrial integration in central China. Secondly, industrial extension and human capital have a significant negative impact relationship on the efficiency of rural industrial integration in central China. Among them, the industrial extension has a significant negative impact on the efficiency of rural industrial integration in central China, because the proportion of primary industry in the industrial structure of central China is larger, and when the increase of primary industry accounts for a higher proportion of the regional GDP, it laterally reflects the relative homogeneity in the industrial structure of the region and the over-dependence on agriculture, which restricts the integration of the various industries in rural areas in the central region. In addition, it is possible that capital and technology in central China are mainly invested in the primary industry, resulting in insufficient capital and technology being invested in the secondary and tertiary industries, thus reducing the efficiency of rural industrial integration in central China; Human capital has a significant negative impact on the efficiency of rural industrial integration in central China, because although overall rural per capita years of schooling in central China are on the rise, the mismatch between the educational structure and the needs of rural industrial integration result in educational achievements that do not be transformed into actual productivity. Thirdly, industrial structure and consumption level have no effect on the efficiency of rural industrial integration in central China. Among them, industrial structure has no effect on the efficiency of rural industrial integration in central China, because of the effect of the difference between urban and rural industrial structure, that is, the industrial structure of cities in central China is more diversified, while rural areas are mostly dominated by traditional agriculture, which makes it difficult for rural areas to directly benefit from the development of the tertiary industry in urban areas, thus restricting the improvement of the efficiency of rural industrial integration in central China; Consumption level has no effect on the efficiency of rural industrial integration in central China, because of the income gap and consumption stratification, with fewer relatively affluent rural residents and more relatively poor rural residents, resulting in a consumption demand that cannot support the development of rural industrial integration, which will reduce the efficiency of rural industrial integration in central China.

In the western region, firstly, the consumption level has a significant positive impact on the efficiency of rural industrial integration in western China, because in the context of the escalating consumption level, the state has increased the provision of policy support for the integration of rural industries in the west as well as the rural areas in the west have their own unique natural resources and humanities, which makes the efficiency of the integration of rural industries in the western region of China greatly improved. Secondly, industrial extension and infrastructure have a significant negative impact on the efficiency of rural industrial integration in western China. Among them, industrial extension has a significant negative impact on the efficiency of rural industrial integration in western China, because the overdevelopment of the primary industry squeezes the space for the development of the secondary and tertiary industries, which is an important support for rural industrial integration, and its lagging development reduces the efficiency of rural integration; infrastructure has a significant negative impact relationship on the efficiency of rural industrial integration in western China, because of the inefficiency of infrastructure investment in the western region, which has not been effectively transformed into a driving force for rural industrial integration. Thirdly, industrial structure and human capital have no effect on the efficiency of rural industrial integration in western China. Among them, the industrial structure has no effect on the efficiency of rural industrial integration in western China, because the western region is affected by historical, ethnic, economic and geographic factors, and even though the value added of the tertiary industry as a percentage of regional GDP has increased, the level of development of the tertiary industry has not yet reached the critical point for effectively improving the efficiency of rural industrial integration; Human capital has no effect on the efficiency of rural industrial integration in western China, because there are problems with the household registration system and the imperfect social security system in the western region, which limit the movement of the rural population to the city, and at the same time impede the movement of high-quality urban talents to the rural areas, and this two-way mobility obstacle restricts the role of human capital in improving the efficiency of rural industrial integration.

**Robustness Tests.** In order to ensure the stability of the results of the research on the factors affecting the efficiency of rural industrial integration, the method of adding explanatory variables was used to conduct a robustness test, as shown in Table 9.

**Table 9:** Robustness test results for eastern, central and western China.

Variable	East	Central	West
lnIS	7.582** (4.911)	0.035 (0.914)	−0.016 (−0.206)
lnIE	0.181 (0.810)	−0.048** (−3.408)	−0.081** (−4.254)
lnINF	2.507* (2.040)	0.095* (2.380)	−0.232** (−5.716)
lnHC	−15.140** (−4.329)	−0.170* (−1.987)	0.077 (1.474)
lnCL	1.613* (2.568)	−0.027 (−1.414)	0.078** (2.924)
lnSII	−0.298 (−0.780)	−0.004 (−0.317)	0.026 (1.890)
_Cons	−27.575** (−4.490)	0.092 (0.463)	0.614* (2.107)
Log(Sigma)	0.280** (4.351)	−3.601** (−47.772)	0.614* (2.107)
N	121	88	132
Likelihood ratio test	X^2^ (6)=75.470,p = 0.000	X^2^ (6)=37.044,p = 0.000	X^2^ (6)=45.512,p = 0.000

By adding explanatory variables (service sector integration is denoted by SII), the results still support the results of the original regression, indicating that the findings of this paper are robust.

## Discussion

### Research innovation

The contributions of this paper are as follows: Firstly, we measured the rural industrial integration efficiency across 31 provinces (municipalities, and autonomous regions) in China from 2012 to 2022. This included comparing the overall efficiency, pure technical efficiency, and scale efficiency of 11 provinces (municipalities, and autonomous regions) in the eastern region, 8 in the central region, and 12 in the western region. This approach effectively avoids the meaningless comparisons that could arise from the different starting points of rural industrial integration efficiency in the east, central, and west. Secondly, the application of the non-expected non-angles super-efficiency EBM model to study the efficiency of rural industrial integration effectively avoids the issue of calculating many provinces’ rural industrial integration efficiency as 1, which prevents further comparative analysis. Thirdly, conduct a study on the influencing factors of the efficiency of rural industry integration in the eastern, central, and western regions of China, comparing the different impacts of various influencing factors on different regions in a more targeted manner.

## Conclusion

Using the non-expected non-angle super-efficiency EBM model, the rural industrial integration efficiency of 31 Chinese provinces (municipalities and autonomous regions) in the east, central and west of China from 2012 to 2022 was measured separately, the Dagum Gini coefficient was used to measure and decompose the degree of spatial differentiation of rural industrial integration efficiency, the kernel density estimation method was used to reveal the dynamic distribution characteristics of rural industrial integration efficiency, and the Tobit model was used to analyze the influencing factors of rural industrial integration efficiency. Tobit model was used to analyze the influencing factors of rural industrial integration efficiency, and the following main conclusions were drawn:

Firstly, from 2012 to 2022, the combined efficiency and pure technical efficiency of rural industrial integration in the east show a rapid growth trend, while the scale efficiency of rural industrial integration in the east shows a decreasing trend; At present, pure technical efficiency is the core driver of the efficiency of rural industrial integration in eastern China, and deepening the scale efficiency of rural industrial integration in eastern China is a top priority for future work. The combined efficiency, pure technical efficiency and scale efficiency of rural industrial integration in central China are all on an increasing trend; At present, the combined efficiency is the core driver of the efficiency of rural industrial integration in central China, and deepening the efficiency of the scale of rural industrial integration in central China is a top priority for future work. The combined efficiency and scale efficiency of rural industrial integration in the west are both on a downward trend, while the pure technical efficiency of rural industrial integration in the west is on an upward trend; At present, pure technical efficiency is the core driver of the efficiency of rural industrial integration in western China, and deepening the efficiency of the scale of rural industrial integration in western China is a top priority for future work.

Secondly, in terms of the degree of spatial differentiation of intra-regional and inter-regional rural industrial integration efficiency, the intra-regional Dagum Gini coefficients of rural industrial integration efficiency in eastern and central China generally show a decreasing trend from 2012 to 2022, indicating that the spatial imbalance type of rural industrial integration efficiency within the eastern and central regions of China is weakening; The intra-regional Dagum Gini coefficient of rural industrial integration efficiency in western China is generally on an upward trend, indicating that the spatial imbalance type of rural industrial integration efficiency within the western China region has increased. The Dagum Gini coefficient of the efficiency of rural industrial integration between eastern and central China, and between eastern and western China, generally shows a decreasing trend, indicating a narrowing of the gap in the efficiency of rural industrial integration between eastern and central China, and between eastern and western China; The Dagum Gini coefficient of rural industrial integration efficiency in central and western China shows an overall upward trend, indicating that the gap in rural industrial integration efficiency between central and western China has intensified. In terms of the contribution of the sources of spatial variation, the current contribution of the sources of spatial differentiation in the efficiency of rural industrial integration in China is, in order, hyper-variable density > inter-regional differences > intra-regional differences.

Thirdly, the distribution curves of three-dimensional kernel density in China’s eastern, central and western regions show a multi-peak trend from 2012 to 2022, indicating that there is a trend of differentiation in the efficiency of rural industrial integration among China’s eastern, central and western regions, and that there is a serious differentiation in the efficiency of rural industrial integration in the eastern region. The ductile shrinkage of the primary and secondary peaks of the three-dimensional kernel density distribution curve in central China from 2016 to 2022 indicates that although there is still a clustering trend in the efficiency of rural industrial integration in central China, the gap in the efficiency of rural industrial integration among central regions is on a narrowing trend, which contributes to the common development of rural industrial integration in central China. The main peak of the three-dimensional kernel density distribution curve narrows in ductility from 2012 to 2022, indicating that the gap in the efficiency of rural industrial integration is narrowing across provinces (municipalities and autonomous regions) in western China.

Lastly, in terms of the influencing factors of rural industrial integration efficiency, in the eastern region, the industrial structure, infrastructure and consumption level in eastern China have a significant positive impact on the efficiency of rural industrial integration in eastern China. Human capital has a significant negative impact on the efficiency of rural industrial integration in eastern China. The industrial extension has no impact on the efficiency of rural industrial integration in eastern China. In the central region, infrastructure has a significant positive impact on the efficiency of rural industrial integration in central China. Industrial extension and human capital have a significant negative impact on the efficiency of rural industrial integration in central China. The industrial structure and consumption level have no impact on the efficiency of rural industrial integration in central China. In the western region, the consumption level has a significant positive impact on the efficiency of rural industrial integration in western China. Industrial extension and infrastructure have a significant negative impact on the efficiency of rural industrial integration in western China. The industrial structure and human capital have no impact on the efficiency of rural industrial integration in western China.

### Limitations and future research directions

The limitations of the study and future research directions are as follows. First, China’s vast territory, the level of economic development in different regions, resource endowment conditions, social resources, etc., there are large differences, although this paper subregional research on the efficiency of rural industrial integration, but different regions to implement the development of rural industrial integration of countermeasures also has distinctive geographical characteristics, follow-up need to field study of the efficiency of integration of rural industries in different regions of China, and more targeted to put forward the specific development of each region. Second, due to the limited data that can be collected at present, which leads to certain limitations in terms of the construction of the indicator system, the follow-up will continue to follow up on the collection of relevant data to further analyze the efficiency of the development of the integration of rural industries and to deeply analyze the reasons behind it.

### Future prospects

Firstly, the structure of financial inputs for rural industrial integration should be strengthened. Each region in China optimizes the structure of capital investment in land, labor, capital, machinery and fertilizer according to local conditions, focuses on the integration and optimization of the industrial chain, improves the efficiency of the use of capital for China’s rural industrial integration, and avoids low-level duplication and waste. For example, it fully exploits the potential for scale efficiency in rural industrial integration in China’s eastern, central and western regions, and strives to combine scientific planning, precise inputs and optimal outputs; The government should set up relevant special funds and formulate tax incentives, and in particular, the government should give strong support to projects that are exemplary and innovative; Financial institutions should expand the scale of rural credit and reduce financing costs to provide more low-cost funds for farmers to participate in the construction of China’s rural industrial integration.

Secondly, the establishment of a system for evaluating the efficiency of integrated rural industrial development. Firstly, the assessment objectives should be clearly defined. The objectives of the integrated development of rural industries are mainly farmers’ profitability, agricultural income, rural prosperity and ecological greenery. Secondly, scientific, comprehensive and systematic assessment indicators for the efficiency of integrated development of rural industries should be formulated according to the assessment objectives. For example, the level of farmers’ income and agricultural output per unit area. Then, develop specific assessment processes and methods for rural industrial integration. A third-party assessment team for the efficiency of rural industrial integration development will be established to truly and objectively evaluate the implementation of the efficiency of rural industrial integration development. In addition, incentive mechanisms are established. For example, the results of the assessment should be used as an important measure for the promotion of the personnel concerned, thus increasing the conscientiousness and motivation of those responsible. Finally, supervision and feedback should be strengthened. A special monitoring body should be set up and assessment results should be published regularly to ensure fairness and impartiality; at the same time, a feedback platform for the integration of rural industries should be set up to collect opinions and suggestions in a timely manner, so as to improve and optimize the assessment system on an ongoing basis.

Thirdly, improvement of rural infrastructure and strengthening of public services. Firstly, improving rural infrastructure. For example, improving rural transportation facilities to ensure the smooth flow of agricultural products and means of production, while facilitating travel for farmers; improvement of water conservancy facilities and strengthening of rural drinking water safety projects to improve the efficiency of irrigation and drainage of farmland, while providing guarantees for the safety of farmers’ drinking water; improve rural communication facilities, increase the coverage of broadband networks and raise the level of rural informatization. Secondly, public services should be strengthened. For example, strengthening education services, increasing investment in rural education, and improving the quality of rural schooling and education; Strengthening cultural construction services. Libraries and cultural activity centers have been built to enrich the spiritual culture of farmers, while various cultural activities have been organized to tell local stories and promote rural cultural heritage and development; Strengthening medical construction services. Improving the rural medical service system in order to enhance the service capacity and level of rural medical institutions; Strengthening the training and introduction of medical personnel in order to enhance the capacity and quality of rural medical services. Lastly, the construction of rural infrastructure and public services should be planned in an integrated manner. Full consideration is being given to the needs of rural infrastructure and public service construction, and existing resources are being efficiently utilized to ensure that both are coordinated with the integrated development of China’s rural industries.
